# The Mediating Role of Social Support on the Relationship Between Alexithymia and Internet Addiction Among Jordanian University Students: A Cross-Sectional Study

**DOI:** 10.3390/ijerph23060748

**Published:** 2026-06-02

**Authors:** Shaher H. Hamaideh, Abdallah Abu Khait, Sawsan Abuhammad, Hanan Al-Modallal, Ayman Hamdan-Mansour, Rami Masa’deh, Mohammed ALBashtawy

**Affiliations:** 1 Community and Mental Health Nursing Department, Faculty of Nursing, The Hashemite University, Zarqa 13133, Jordan; 2 Department of Nursing, Collage of Heath Sciences, University of Sharjah, Sharjah P.O. Box 27272, United Arab Emirates; 3 Department of Maternal and Child Health, Faculty of Nursing, Jordan University of Science and Technology, Irbid 22110, Jordan; 4 Community Health Department, School of Nursing, The University of Jordan, Amman 11942, Jordan; 5 Nursing Department, Faculty of Nursing, Applied Science Private University, Amman 11973, Jordan; 6 Princess Salma Faculty of Nursing, Al Al-Bayt University, Mafraq 25113, Jordan

**Keywords:** alexithymia, internet addiction, social support, university students, Jordan

## Abstract

**Highlights:**

**Public health relevance—How does this work relate to a public health issue?**

- Alexithymia and Internet addiction are emerging psychological and public health concerns among university students.
- University students use electronic technologies and screens extensively, which may increase vulnerability to reduced face-to-face communication and problematic Internet use.

**Public health significance—Why is this work of significance to public health?**

- The overall mean scores for alexithymia, Internet addiction, and social support indicate a need for early identification and prevention strategies.
- Significant associations were observed among alexithymia, social support, and Internet addiction.

**Public health implications—What are the key implications or messages for practitioners, policy makers and/or researchers in public health?**

- Universities should pay attention to students who are at risk of alexithymia and Internet addiction, especially with the absence of social support.
- Practitioners, policy makers, and researchers in public health should develop interventions targeted to the prevention of alexithymia and internet addiction, and strengthen social support systems.

**Abstract:**

Background: Alexithymia and Internet addiction are emerging concerns among university students, and perceived social support may help explain how difficulties in identifying and expressing emotions relate to problematic Internet use. This study examined the mediating role of perceived social support in the relationship between alexithymia and Internet addiction among Jordanian university students. Methods: A descriptive cross-sectional online survey was conducted among 300 university students in Jordan during the 2022/2023 academic year using Google Forms distributed through Facebook and Microsoft Teams. Participants completed the Toronto Alexithymia Scale (TAS-20), Internet Addiction Test (IAT), Multidimensional Scale of Perceived Social Support (MSPSS), and demographic questions. Data were analyzed using descriptive statistics, Pearson correlations, multiple regression, and Hayes PROCESS macro Model 4 with 5000 bootstrap samples. Results: The overall mean scores for alexithymia, Internet addiction, and social support were 62.57, 46.05, and 55.13, respectively. Alexithymia was positively correlated with Internet addiction and negatively correlated with social support. Social support partially mediated the relationship between alexithymia and Internet addiction, indicating that higher alexithymia was associated with lower perceived social support, which in turn was associated with higher Internet addiction. Conclusions: The findings support the need for university-based screening and prevention programs that address emotional awareness, healthy Internet use, and social support. Theoretically, the results suggest that social support is a meaningful psychosocial pathway linking alexithymia with problematic Internet use among university students.

## 1. Introduction

Alexithymia is a personality-related construct characterized by difficulty identifying feelings, difficulty describing feelings, and an externally oriented thinking style [1,2]. These difficulties can impair emotional regulation and interpersonal communication, thereby increasing vulnerability to psychological distress and behavioral addictions, including problematic Internet use [3,4,5,6]. Among university students, alexithymia is particularly relevant because the transition to higher education involves academic pressure, new social roles, greater autonomy, and increased reliance on digital communication.

Previous studies have reported variable rates of alexithymia across non-clinical and student populations, with estimates influenced by age, culture, language version, sampling strategy, and the TAS-20 cut-off used [7,8,9,10,11,12]. Because Jordanian university students study in a sociocultural context where emotional expression, family connectedness, and social expectations may shape help-seeking and communication, updated evidence is needed to clarify the level and correlates of alexithymia in this population.

Internet access is now embedded in students’ academic and social lives. While Internet use is not inherently harmful, uncontrolled or excessive use can be associated with psychological distress, lower self-esteem, social isolation, reduced perceived social support, and Internet addiction [13,14,15,16,17]. Recent international evidence indicates that problematic Internet-related behaviors remain common among university students and have increased in importance since the COVID-19 period [18]. Related research on cyberloafing and smartphone addiction also suggests that digital overuse among students should be understood within broader academic stress and maladaptive coping frameworks [19,20].

University students may be vulnerable to Internet addiction because of easy access to smartphones, online learning platforms, social media, and limited external monitoring [21,22]. In Jordan, Al-Gamal et al. reported Internet addiction among university students using the Internet Addiction Test and a defined cut-off score, whereas Samaha et al. reported moderate-to-severe Internet addiction among Lebanese college students using IAT classifications [23,24]. These prevalence estimates should therefore be interpreted in light of the instruments, thresholds, and study contexts used in each study.

In the Jordanian university context, problematic Internet use should be interpreted alongside students’ academic demands, online learning practices, and family- and peer-oriented social norms. Perceived social support is therefore especially relevant because students may depend on family, friends, and significant others for emotional disclosure, coping, and help-seeking when facing academic or psychological stress [20,23,25].

Perceived social support refers to the extent to which individuals believe that family members, friends, and significant others are available to provide emotional and practical help when needed [26]. Adequate social support is associated with better psychological health, resilience, academic motivation, self-esteem, and coping, while insufficient support is associated with stress, loneliness, anxiety, depression, alexithymia, and problematic Internet use [14,27,28,29].

Theoretically, alexithymia may contribute to Internet addiction because students who struggle to identify and communicate emotions may prefer online interaction, where communication can feel more controllable and less emotionally demanding [10,30,31,32,33,34]. Social support may weaken this pathway by providing students with opportunities to express emotions, receive feedback, and cope with stress offline [35,36]. Accordingly, social support was examined as a mediator between alexithymia and Internet addiction.

From a stress-coping perspective, social support can function as a psychosocial resource that reduces reliance on avoidant or compensatory online behaviors. Testing social support as a mediator helps explain how emotional-expression difficulties may translate into problematic Internet use through reduced perceived interpersonal resources [14,26,27,28,29,35,36].

### Significance and Research Questions

Although relationships among alexithymia, social support, and Internet addiction have been reported in prior studies, recent evidence among Jordanian university students remains limited. This study therefore examined the levels of alexithymia, Internet addiction, and perceived social support and tested a mediation model in which social support explains part of the association between alexithymia and Internet addiction. Based on previous literature, the study tested the following hypotheses: The empirical gap is particularly important in Jordan, where recent student-focused evidence remains limited and existing regional studies have used different samples, instruments, and thresholds. This study contributes by testing a theoretically grounded mediation model in a Jordanian university sample rather than examining only bivariate associations.

Recent Jordanian nursing and health-science education studies further support the importance of strengthening evidence-based practice in clinical settings [37]. In related student populations, stress-reduction interventions such as guided imagery and progressive muscle relaxation have reduced physical and emotional symptoms during initial clinical training [38], and mental health coursework has improved nursing students’ attitudes toward seeking professional psychological help [38].

**H1.**  *Alexithymia is positively associated with Internet addiction among Jordanian university students.*

**H2.**  *Alexithymia and Internet addiction are negatively associated with perceived social support.*

**H3.**  *Selected demographic and psychosocial variables significantly predict alexithymia, Internet addiction, and perceived social support.*

**H4.**  *Perceived social support mediates the relationship between alexithymia and Internet addiction.*

## 2. Methods

### 2.1. Design

A descriptive, cross-sectional design was used to assess the mediating role of perceived social support in the relationship between alexithymia and Internet addiction among Jordanian university students.

### 2.2. Participants

A convenience sampling strategy was used. The target population was Jordanian undergraduate students enrolled in public and private universities. To estimate the required sample size, G*Power version 3 was used with a medium effect size of 0.15, power of 0.80, alpha of 0.05, and a planned maximum set of 15 potential predictor variables: age, gender, sleeping hours, smoking status, weekly Internet-use hours, GPA, year of study, faculty major, university type, monthly family income, bedroom-sharing status, region, perceived physical health, perceived psychological health, and life satisfaction. The minimum required sample size was 139; the final sample included 300 students, which exceeded this requirement. The sample-size calculation reflected the largest planned predictor set, whereas the final regression tables present only the statistically significant predictors retained in each model.

### 2.3. Measures

The questionnaire consisted of four parts: demographic variables, Toronto Alexithymia Scale (TAS-20), Internet Addiction Test (IAT), and Multidimensional Scale of Perceived Social Support (MSPSS).

Demographic variables were: age, gender, sleeping hours, smoking status, weekly hours spent on the Internet, GPA (fair, good, very good, or excellent), year of study, faculty major (humanistic, scientific, or health), university type (public or private), monthly family income, bedroom-sharing status, university region, perceived physical health, perceived psychological health, and life satisfaction.

Perceived physical health, perceived psychological health, and life satisfaction were assessed using single items rated from 1 (very poor/dissatisfied) to 5 (very strong/satisfied). Smoking status and life satisfaction were included because previous evidence links health behaviors and subjective well-being with behavioral addictions and perceived social support; therefore, these variables were examined as potential predictors in the regression analyses.

Toronto Alexithymia Scale (TAS-20) [39] was used to measure alexithymia. The TAS-20 consists of 20 items divided into three subscales: difficulty identifying feelings (DIF, 7 items), difficulty describing feelings (DDF, 5 items), and externally oriented thinking (EOT, 8 items). Items are rated on a 5-point Likert scale ranging from 1 (strongly disagree) to 5 (strongly agree), with total scores ranging from 20 to 100. Scores above 60 indicate alexithymia, scores from 52 to 60 indicate possible alexithymia, and scores of 51 or below indicate no alexithymia [2]. The scale has demonstrated acceptable reliability and validity [39,40]. In the current study, Cronbach’s alpha was 0.853 for the total TAS-20, 0.890 for DIF, 0.703 for DDF, and 0.515 for EOT.

Internet Addiction Test (IAT) [41] was used to measure Internet addiction. The IAT consists of 20 items assessing preoccupation, compulsive use, behavioral problems, emotional changes, and life interference related to Internet use. Each item is rated from 1 (rarely) to 5 (always), and total scores range from 20 to 100. Scores below 50 indicate normal Internet use, scores from 50 to 79 indicate moderate Internet addiction, and scores from 80 to 100 indicate severe Internet addiction [41,42]. The IAT was analyzed as a total scale because no subscale scores were used in the present study. In the current study, Cronbach’s alpha for the total IAT was 0.958.

Multidimensional Scale of Perceived Social Support (MSPSS) [43] was used to measure perceived social support. The MSPSS consists of 12 items grouped into three subscales: family, friends, and significant others. Items are rated from 1 (very strongly disagree) to 7 (very strongly agree), and total scores range from 12 to 84, with higher scores indicating greater perceived social support. Subscale and total scores can also be interpreted by item mean: 1.0 to <3.0 indicates low support, 3.0 to 5.0 indicates moderate support, and >5.0 to 7.0 indicates high support [43]. The MSPSS has demonstrated good reliability and validity [44]. In the current study, Cronbach’s alpha was 0.920 for the total MSPSS, 0.863 for family support, 0.918 for friend support, and 0.896 for support from significant others.

### 2.4. Procedures and Ethical Considerations

Data were collected during the 2022/2023 academic year using an anonymous online questionnaire created in Google Forms. The survey link was distributed through student electronic groups on Facebook and Microsoft Teams. Because the survey link was distributed through open online channels, the exact denominator of students who viewed the invitation could not be determined and a response rate could not be calculated. This recruitment approach may have increased the participation of students who were active on social media or online learning platforms and may partly explain the uneven gender and university-type distribution in the sample.

All ethical considerations were assured before, during, and after conducting the study. Approval to conduct the study was obtained from the Institutional Review Board of The Hashemite University (No. 61/1/2022/2023). An additional IRB approval/renewal was obtained on 16 April 2025 (No. 47/3/2024/2025). The study purpose, voluntary nature of participation, confidentiality, anonymity, and the right to withdraw without penalty were explained on the first page of the online questionnaire. Participants provided informed consent electronically by proceeding to and submitting the questionnaire.

### 2.5. Data Analysis

Data were analyzed using IBM SPSS Statistics version 28 and PROCESS macro v4.2 by Andrew F. Hayes [45]. Data screening included checking for out-of-range values, duplicate submissions, and missing item responses before calculating total scale and subscale scores. Questionnaires with incomplete scale data were not retained in the final analytic dataset; therefore, all analyses were conducted using 300 complete cases. Descriptive statistics, including frequencies, percentages, means, ranges, and standard deviations, were used to describe demographic variables, alexithymia, Internet addiction, and social support. Pearson correlation coefficients were used to examine associations among alexithymia, Internet addiction, and social support. Multiple regression analyses were used to identify predictors of alexithymia, Internet addiction, and social support. The mediation effect was tested using PROCESS Model 4 with alexithymia as the independent variable, Internet addiction as the dependent variable, and social support as the mediator. No covariates were included in the mediation analysis. The indirect effect was tested using 5000 bootstrap samples and 95% bootstrap confidence intervals. Statistical significance was set at alpha = 0.05.

## 3. Results

### 3.1. Demographic Characteristics

The sample consisted of 300 participants; 72 (24.0%) were male and 228 (76.0%) were female. Participants’ mean age was 20.46 years (SD = 2.10). Most participants were non-smokers (*n* = 236, 78.7%) and were enrolled in public universities (*n* = 279, 93.0%). Participants spent an average of 33.14 h per week on the Internet. Although this mean indicates frequent Internet use, Internet addiction was classified using IAT cut-off scores rather than Internet-use hours alone. Participants reported adequate perceived physical and psychological health and adequate relationships with parents, friends, and family members. Other demographic variables are shown in Table 1.

### 3.2. Levels and Correlations of Alexithymia, Internet Addiction, and Social Support

The overall mean scores for alexithymia, Internet addiction, and social support were 62.57, 46.05, and 55.13, respectively. Regarding alexithymia subscales, the highest mean score was for EOT (M = 3.31 out of 5, SD = 0.535), followed by DDF (M = 3.03, SD = 0.798) and DIF (M = 2.99, SD = 0.931). The prevalence of alexithymia was 36.6% using the study classification reported in Table 2. For Internet addiction, most participants used the Internet normally (*n* = 199, 66.3%), 25.0% used it moderately, and 8.7% used it severely. Participants considered Internet addicted, based on IAT scores of 50 or more, were 101 (33.7%). Regarding social support, the highest support came from family, followed by significant others and friends (Table 2).

Table 3 shows the relationships among the studied variables. Alexithymia correlated positively with Internet addiction (r = 0.333, *p* = 0.001) and negatively with social support (r = −0.286, *p* = 0.001). Internet addiction correlated negatively with social support (r = −0.325, *p* = 0.001).

### 3.3. Predictors of Alexithymia, Internet Addiction, and Social Support

Three separate multiple regression analyses were performed. Variables that predicted alexithymia were perceived physical health, perceived psychological health, and Internet addiction. Variables that predicted Internet addiction were age, smoking status, income, social support, and alexithymia. Because smoking was coded as 1 = yes and 2 = no, the negative coefficient indicates that smokers reported higher Internet addiction scores than non-smokers. Variables that predicted social support were income, life satisfaction, and Internet addiction. The corrected t-values, *p*-values, and 95% confidence intervals are shown in Table 4.

### 3.4. The Mediation Effect of Social Support

**Figure 1:**
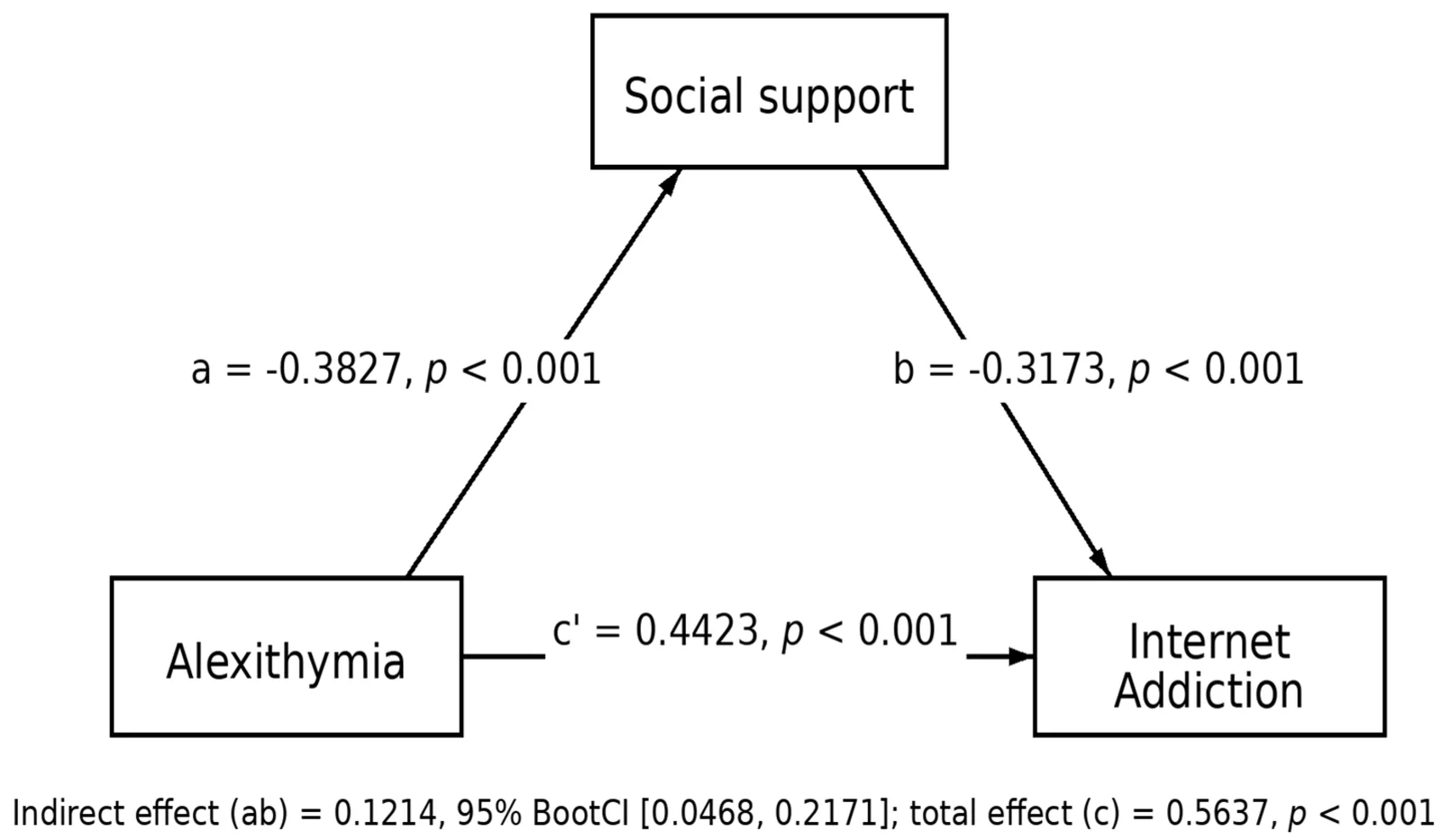
Results of the Mediation Model. Note. a = effect of alexithymia on social support; b = effect of social support on Internet addiction controlling for alexithymia; c′ = direct effect; ab = indirect effect.

The mediation analysis assessed the mediating role of perceived social support in the relationship between alexithymia and Internet addiction. PROCESS Model 4 showed that alexithymia significantly predicted social support (path a: b = −0.3827, SE = 0.0743, t = −5.1502, *p* < 0.001), and social support significantly predicted Internet addiction while controlling for alexithymia (path b: b = −0.3173, SE = 0.0699, t = −4.5417, *p* < 0.001). The total effect of alexithymia on Internet addiction was significant (path c: b = 0.5637, SE = 0.0925, t = 6.0938, *p* < 0.001), and the direct effect remained significant after including the mediator (path c′: b = 0.4423, SE = 0.0935, t = 4.7303, *p* < 0.001). The indirect effect was significant because the 95% bootstrap confidence interval did not include zero (ab = 0.1214, BootSE = 0.0430, BootLLCI = 0.0468, BootULCI = 0.2171). The completely standardized indirect effect was 0.0717 (BootSE = 0.0240, BootLLCI = 0.0290, BootULCI = 0.1231). Thus, social support partially mediated the relationship between alexithymia and Internet addiction. Mediation results are presented in Table 5 and Figure 1.

## 4. Discussion

The current study found a relatively high mean alexithymia score among Jordanian university students. This finding is higher than many studies conducted in comparable student samples [3,4,5,6,9,12,25,36,46,47,48]. Several explanations are possible. First, the online recruitment strategy may have reached students who were more digitally active and potentially more socially withdrawn. Second, cultural norms regarding emotional expression may influence how students identify and describe feelings. Third, the sample included a high proportion of female students and students from public universities, which may limit direct comparison with more balanced samples.

The prevalence of alexithymia was also higher than most comparable studies, which have reported rates ranging from 13.5% [30] to 30.2% [31]. In similar Jordanian research, Hamaideh [25] reported a lower prevalence among university students. Differences between studies may reflect measurement timing, cut-off scores, sample characteristics, academic stress, and the increased reliance on online communication after the COVID-19 period. Therefore, this result should be interpreted as evidence of a potentially important public health concern rather than as a nationally representative estimate.

The mean Internet addiction score was 46.05, and 33.7% of participants met the IAT threshold for moderate or severe Internet addiction. This prevalence is lower than some reports in international student samples but remains clinically and educationally meaningful [9,18,49]. The finding is also consistent with recent regional concerns about problematic technology use among university students, including smartphone addiction and related psychological distress in Jordan [20]. Importantly, the average weekly Internet-use hours should not be interpreted as addiction by itself; addiction classification was based on the IAT cut-off scores, which assess impairment and compulsive patterns rather than time online alone.

Alexithymia was positively correlated with Internet addiction and negatively correlated with social support, while Internet addiction was negatively correlated with social support. These findings are consistent with evidence that students with alexithymic features may rely more heavily on online environments because digital interaction can reduce the immediacy and complexity of emotional expression [12,30,31,32,33,50]. Conversely, students with stronger perceived social support may have more opportunities for offline emotional disclosure, coping, and belonging, which may reduce reliance on problematic Internet use [14,27,28,29,40,47].

The regression analyses showed that perceived physical health, perceived psychological health, and Internet addiction predicted alexithymia. Younger age, smoking status, income, lower social support, and alexithymia predicted Internet addiction. Income, life satisfaction, and Internet addiction predicted perceived social support. These findings suggest that Internet addiction in this sample is embedded in a broader psychosocial pattern involving emotional difficulties, perceived support, health behaviors, and well-being rather than being explained by Internet-use time alone.

The association between smoking status and Internet addiction should be interpreted cautiously. Smoking may indicate broader risk-taking, stress-coping patterns, or social contexts associated with problematic Internet use, but causality cannot be inferred from the cross-sectional design. Similarly, life satisfaction may reflect general well-being that facilitates perceived social support, but reciprocal relationships are also possible.

The mediation model provides a theoretical explanation for the observed relationships. Students with alexithymia may have difficulty identifying and verbalizing emotions, which can interfere with seeking and receiving support from family, friends, and significant others [2]. Lower perceived social support may then increase the likelihood of using the Internet as a compensatory environment for connection, distraction, or emotion regulation. The significant direct effect after adding social support indicates partial mediation, suggesting that other mechanisms, such as loneliness, self-control, depression, anxiety, or maladaptive coping, may also explain the relationship between alexithymia and Internet addiction.

Practically, the findings suggest that interventions should not focus only on reducing screen time. University counseling services and student affairs units may consider programs that strengthen emotional awareness, communication skills, peer support, family connectedness, and adaptive coping. Screening students with elevated alexithymia or low perceived social support may help identify groups at higher risk for problematic Internet use.

These recommendations are consistent with Jordanian nursing education research showing that guided imagery and progressive muscle relaxation can reduce physical and emotional symptoms during initial clinical training [37]. Mental health coursework can also improve students’ attitudes toward seeking professional psychological help [38]. In addition, strengthening research utilization among nurses may support the implementation of evidence-based student-support and health interventions in clinical education settings [51].

### Limitations

The current study has several limitations. First, the cross-sectional design prevents conclusions about causal direction; longitudinal and experimental studies are needed to determine whether alexithymia leads to Internet addiction, whether Internet addiction increases emotional difficulties, or whether these relationships are reciprocal. Second, all variables were measured through self-report questionnaires collected at one time point, which increases the possibility of recall bias, social desirability bias, and common method variance. Because all measures were completed by the same participants using similar response formats, common method bias cannot be completely ruled out. Third, the convenience online sampling strategy through Facebook and Microsoft Teams may have introduced selection bias by over-representing digitally active students. Fourth, the sample was unevenly distributed by gender and university type, with most participants being female and from public universities; therefore, the findings should not be generalized to all Jordanian university students without caution. Fifth, the open online distribution method prevented calculation of a precise response rate. Future studies should use probability or stratified sampling, recruit more balanced samples from public and private universities, include multiple data sources, test additional mediators and moderators, and use longitudinal designs to confirm the temporal sequence of the mediation model.

## 5. Conclusions

This study found meaningful associations among alexithymia, perceived social support, and Internet addiction among Jordanian university students. Alexithymia was positively associated with Internet addiction, whereas social support was negatively associated with both alexithymia and Internet addiction. Social support partially mediated the relationship between alexithymia and Internet addiction, indicating that students with greater difficulty identifying and expressing emotions may be at higher risk for problematic Internet use partly because they perceive less support from others. University-based interventions that combine emotional-awareness training, counseling, peer-support programs, family engagement, and healthy Internet-use education may help reduce risk and promote student well-being.

## Figures and Tables

**Table 1:** Demographic and educational variables (N = 300).

Variable (Range)	Mean	Standard Deviation
Age	20.46	2.10
Average daily sleeping hours	7.78	1.64
Hours spend on Internet/week	33.14	22.37
Family income/month (Jordanian Dinar)	785.00	557.89
Perception of physical health (Scale from 1 to 5)	3.92	0.86
Perception of psychological health (Scale from 1 to 5)	3.36	1.13
Satisfaction in life (Scale from 1 to 5)	3.89	1.04
	Frequency	Percentage
**Gender**		
Male	72	24.0
Female	228	76.0
**Smoking status**		
Yes	64	21.3
No	236	78.7
**Year of study**		
First year	62	20.7
Second year	110	36.7
Third year	73	24.3
Fourth year	51	17.0
Fifth year	4	1.3
**Grade Point Average (GPA)**		
Fair	22	7.3
Good	81	27.0
Very good	120	40.0
Excellent	77	25.7
**Faculties major**		
Humanistic faculty	57	19.0
Scientific faculty	120	40.0
Health faculty	123	41.0
**University type**		
Public	279	93.0
Private	21	7.0
**Staying in bedroom**		
Alone in your bedroom	105	35.0
Shared with family member(s)	195	65.0
**University location**		
North/South	77	25.7
Middle	223	74.3

**Table 2:** Levels and prevalence of alexithymia, Internet addiction, and social support (N = 300).

Variable	Mean	Standard Deviation
**Alexithymia (overall)**	**62.57**	**11.94**
Difficulty identifying feelings (DIF)	20.93	6.52
Difficulty describing feelings (DDF)	15.14	3.99
Externally oriented thinking (EOT)	26.50	4.28
**Internet Addiction (overall)**	**46.05**	**20.22**
Normal use	34.03	8.55
Moderate use	62.34	8.88
Severe use	91.03	5.41
**Social support (overall)**	55.13	15.83
Social support from family	19.02	5.94
Social support from friends	17.45	6.39
Social support from significant others	18.66	6.53
	Frequency	Percentages
**Internet Addiction**		
Normal use (20–49)	199	66.3
Moderate use (50–79)	75	25.0
Severe use (80–100)	26	8.7
**Alexithymia**		
Normal level (20–51)	70	23.3
Possible alexithymia (52–60)	120	40.0
Alexithymia (61–100)	110	36.7

**Table 3:** Mean scores and correlations of alexithymia, Internet addiction, and social support (N = 300).

Variable	1	2	3
1. Alexithymia	1.00		
2. Internet Addiction	0.333 **	1.00	
3. Social Support	−0.286 **	−0.325 **	1.00
Mean	62.57	46.05	55.13
Standard Deviation	11.94	20.22	15.83

** Significance at an alpha of 0.01 (two-tailed).

**Table 4:** Multiple regression models predicting alexithymia, Internet addiction, and social support (N = 300).

	B	β	*t*	*p*		
**Predictors of Alexithymia**					Lower	Upper
1. Perception of physical health	−1.93	−0.140	−2.23	0.026	−3.63	−0.23
2. Perception of mental health	−2.57	−0.246	−3.35	0.001	−4.08	−1.06
3. Internet addiction	0.197	0.334	5.26	<0.001	0.123	0.271
Model Summary						
R^2^	0.252					
Adjusted R^2^	0.218					
Total Variance	21.8%					
**Predictors of Internet addiction**						
1. Age	−1.308	−0.136	−2.91	0.004	−2.19	−0.42
2. Smoking	−17.008	−0.354	−6.80	<0.001	−21.93	−12.09
3. Income	0.006	0.179	3.65	<0.001	0.003	0.009
4. Social support	−0.321	−0.253	−5.10	<0.001	−0.445	−0.197
5. Alexithymia	0.450	0.266	5.26	<0.001	0.282	0.618
Model Summary						
R^2^	0.399					
Adjusted R^2^	0.373					
Total Variance	37.3%					
**Predictors of Social Support**						
1. Income	0.003	0.109	1.97	0.050	0.000	0.006
2. Life satisfaction	2.503	0.163	2.41	0.017	0.459	4.547
3. Internet addiction	−0.251	−0.317	−5.01	<0.001	−0.350	−0.152
Model Summary						
R^2^	0.247					
Adjusted R^2^	0.216					
Total Variance	21.6%					

**Table 5:** Total, direct, and indirect effects of mediation analysis summary.

Standardized Effect	95% CI	*p*	t	SE/BootSE	b/Effect	Effect
0.3329	0.3816, 0.7457	<0.001	6.0938	0.0925	0.5637	Total effect
0.2612	0.2583, 0.6263	<0.001	4.7303	0.0935	0.4423	Direct effect
0.0717	0.0468, 0.2171	N/A	N/A	0.0430	0.1214	Indirect effect
0.0717	0.0290, 0.1231	N/A	N/A	0.0240	0.0717	Standardized indirect

CI: confidence interval; bootstrap confidence intervals are reported for indirect effects. N/A: not provided by PROCESS for bootstrap indirect effects.

## Data Availability

The data presented in this study is available on request from the corresponding author. The data is not publicly available due to ethical restrictions.
